# A multidimensional investigation of the relationship between skin-mediated somatosensory signals, emotion regulation and behavior problems in autistic children

**DOI:** 10.3389/fnins.2023.1227173

**Published:** 2023-08-10

**Authors:** Inmaculada Riquelme, Samar M. Hatem, Álvaro Sabater-Gárriz, Pedro Montoya

**Affiliations:** ^1^Research Institute on Health Sciences (IUNICS-IdISBa), University of the Balearic Islands, Palma de Mallorca, Spain; ^2^Department of Nursing and Physiotherapy, University of the Balearic Islands, Palma de Mallorca, Spain; ^3^Faculty of Medicine, STIMULUS Research Group (reSearch and TeachIng neuroModULation Uz bruSsel), Vrije Universiteit Brussel, Brussels, Belgium; ^4^Institute of Neuroscience, Université catholique de Louvain, Brussels, Belgium; ^5^Balearic ASPACE Foundation, Marratxí, Spain; ^6^Center for Mathematics, Computation and Cognition, Federal University of ABC, São Bernardo do Campo, Brazil

**Keywords:** autism, somatosensory, emotion, behavior, children

## Abstract

**Introduction:**

Autistic children may have abnormal sensory perception, emotion dysregulation and behavior problems. The aim of this cross-sectional study was to explore the relationship between skin-mediated somatosensory signals and emotion/behavior difficulties in autistic children and adolescents, in comparison typically developing peers (TDP).

**Methods:**

Thirty-eight autistic children and adolescents and 34 TDP completed a multidimensional assessment consisting of the measurement of somatosensory thresholds of touch, pain and temperature, a task on emotion knowledge and parent-reported questionnaires on sensory reactivity, emotion regulation and behavior.

**Results:**

Autistic children had higher pain sensitivity, less sensory reactive behaviors and more behavior problems than their TDP. In contrast to TDP, several somatosensory thresholds of autistic children correlated with emotion regulation and behavior problems.

**Discussion:**

Sensory dysfunction may affect the development of emotional processing and behavior in autistic children and adolescents. This knowledge can lay the foundation for future studies on co-occurring alterations in corresponding neural networks and for the implementation of early interventions, including sensory rehabilitation therapy, for promoting regulated behaviors in autistic children and adolescents.

## 1. Introduction

The interaction between abnormal somatosensory perception, emotion dysregulation and behavior problems in autistic individuals is a topic of recent interest (Green et al., 2016; Will et al., 2019; Fernandez-Prieto et al., 2021). Abnormal reactivity to sensory input is one of the diagnostic criteria for autism spectrum disorders (DSM-5) American Psychiatric Association (2013). Sensory features in autistic individuals may include abnormal sensory perception thresholds, altered response to affective stimuli and lack of embodiment for socio-affective interactions (Masson et al., 2019; Surgent et al., 2022). Atypical sensory behavior, typified as hyper- or hypo-reactivity to sensory stimuli, has been reported in about 75–90% of autistic individuals (Green et al., 2016; Balasco et al., 2020; Kirby et al., 2022). In particular, alterations in somatosensory behaviors may include abnormal tactile discrimination thresholds, altered pain sensitivity, atypical sensory seeking behaviors and tactile aversion (Riquelme et al., 2018; Masson et al., 2019; Buyuktaskin et al., 2021; Espenhahn et al., 2021). Disrupted brain processing of somatosensory stimuli in autistic individuals imply aberrant neural activity in structures such as the brainstem, insula and the thalamocortical network (Failla et al., 2017; Woodward et al., 2017; Surgent et al., 2022). This abnormal brain activity is characterized by general cortical excitability, deficiency of the inhibitory networks, altered filtering, altered discrimination of tactile inputs, altered habituation to tactile inputs and impaired higher-order multisensory integration (Woodward et al., 2017; Sapey-Triomphe et al., 2019; Zetler et al., 2019; Iannone and De Marco García, 2021). Atypical responses to sensory stimuli in autistic individuals have been associated with poor functional outcomes and social competence, thus impacting on quality of life and participation (Roley et al., 2015; Failla et al., 2017; Fabbri-Destro et al., 2022; Surgent et al., 2022).

Autistic children are prone to emotion dysregulation (Rosello et al., 2022). Imaging studies have shown that atypical brain mechanisms impacting structures such as the amygdala and prefrontal cortex, may affect the processing of complex, implicit and social emotional information, such as the recognition of affective faces (Mazefsky et al., 2013; Black et al., 2020). Autistic children may exhibit amplified affective responses and poor emotion regulation (Mazefsky et al., 2013; White et al., 2014) and are at high risk for comorbid mental health problems, such as depression, anxiety and low self-esteem (McCauley et al., 2019; Vasa et al., 2020). Also, three quarters of autistic children show behavior problems (both internalizing and externalizing behavior, such as self-injury, aggression or withdrawal) and poor adaptive functioning (Newcomb and Hagopian, 2018; Cai et al., 2019; Samanta et al., 2022). Behavior problems result in diminished quality-of-life for the autistic individuals and for their families (Newcomb and Hagopian, 2018).

Emotion dysregulation has been suggested as a mediator for the relationship between sensory processing patterns and behavior in autistic individuals (Sung et al., 2022). Problematic emotional states and maladaptive behavior (i.e., aggression or fear) have been associated with a higher odds of having abnormal sensory features (Kirby et al., 2022): anxiety has been associated with sensory over-responsivity (Vasa et al., 2020); and infants with greater fear or shyness presented with higher perceptual sensitivity during childhood (Narvekar et al., 2022). Moreover, a predictive association of sensory difficulties explained more than 50% of the variance of behavior problems (Fabbri-Destro et al., 2022). As an example, auditory hyper-reactivity has been suggested to be responsible of interrupting behavior adaptation in autistic children (Takahashi et al., 2016). Also, mechanosensory processing defects and sensory hyposensitivity may contribute to anxiety-like behavior and social interaction deficits in autistic mouse models (Orefice et al., 2016; Prickard et al., 2020).

Despite the increasing evidence of a relationship between abnormal sensory perception, emotion dysregulation and behavior problems, rehabilitation therapy rarely addresses these problems from an integrated perspective. Examples of rehabilitation treatments addressing a single aspect of this triade are: robotical interventions used for simulating social situations and promoting emotion knowledge; virtual reality and digital applications implemented for training emotion recognition; equine-assisted therapies and physical exercise shown to be effective for improving behavioral functioning in autistic children (Petrovska and Trajkowski, 2019; Tse, 2020; Zhang et al., 2022; Soleiman et al., 2023; Xiao et al., 2023). Similarly, examples of interventions guiding the regulation of sensory strategies are: cognitive behavioral therapy, mindfulness, sensory integration, massage and sensory-adapted environments. These interventions have obtained encouraging results for regulating adverse sensory experiences and enhancing participation (Reis et al., 2021; Yuan et al., 2022; Stein Duker et al., 2023). Nonetheless, novel treatment strategies that aim at modifying sensory perception in order to modulate emotion regulation and behavior, are still scant. From our knowledge, only education on interoception (Mahler et al., 2022) and sensory garments (Lawson et al., 2022) have shown effectiveness both in modifying sensory awareness and improving emotion regulation and participation.

Price and Hooven (2018) defined emotion regulation as «the ability to accurately detect and evaluate cues related to physiological reactions to stressful events, accompanied by appropriate regulation strategies that influence the emotional response». Consequently, the impaired ability to identify and evaluate bodily signals could lead to a deficit in the development of emotional strategies. In its initial sense, interoception was a concept describing how one senses internal signals from one’s body. This concept has developed to encompass all the signals informing from the physiological condition of the body (Craig, 2002), including skin-mediated perception as assessed with psychophysical somatosensory thresholds. Skin-mediated signals such as touch, pain, and temperature are essential for monitoring the body’s physiological state (Crucianelli and Ehrsson, 2023). Though some studies have described the relationship between parent- or self-reported sensory profiles, and emotion and behavior (Sung et al., 2022), there is little data relating psychophysical measurements of somatosensation.

The objective of the present study is to investigate the relationship between skin-mediated somatosensory signals and emotion/behavior difficulties in autistic children and adolescents, in comparison to typically developmental peers (TDP). This study is a necessary preliminary step of laying a solid physiological base for the development of future rehabilitation therapies that aim at normalizing somatosensation in this patient population. For this research, a multidimensional assessment of sensory processing, emotion regulation and behavior problems was used, including psychophysical somatosensory thresholds, parent-reported questionnaires and self-performed tasks. We hypothesize that skin-mediated somatosensation will play an important role in co-occurring alterations in emotion and behavior in autistic children.

## 2. Materials and methods

### 2.1. Participants

Participants with a diagnosis of autism level 1 and 2 according to DMS criteria (DSM5), reported in their medical history by their neurologist, were recruited from patient associations and early care centres in Majorca (Spain) during the year 2022. Inclusion criteria for autism participants were: (Achenbach and Edelbrock, 1983) age between 4 and 16 years and (Allely, 2013) severity levels 1 and 2 of of the DSM-5 (verbal expression at least of simple sentences, requiring low-medium support). Age- and sex-matched typically developing peers, without a diagnosis of autism or other developmental disorders, were also recruited in educational support centres during the same time period.

Potential participants were identified by caregivers from 6 institutions (i.e., 2 patient associations, 4 centres for early-care support) and were sent an advertising letter by the centres’ social media. Interested families transmitted their contact details through an on-line platform.^1^ They were then contacted by a member of the research team by phone or email to provide detailed information about the study. In case parents and children were interested to further participate in the study, they provided their e-mail address to the research team and received in return by e-mail a written information letter, a written consent form and the parent-reported questionnaires, along with the necessary instructions for completion. Furthermore, an appointment was scheduled for the assessment session. The parents brought along the written informed consent and the completed questionnaires at the assessment session. There, the questionnaires were revised face-to-face with parents to solve doubts and avoid blanks. Next, children performed the Emotion Matching Task (EMT) and their somatosensory thresholds were measured by an experienced researcher. With the parents’ and participants’ consent, clinical data (level of severity, verbal ability, the Wechsler Intelligence Scale for Children (WISC) scores, existence of chronic pain, hand dominance) were extracted from the medical and psychological health reports. The presence of chronic pain was double-checked by including a question asking for chronic pain into the questionnaires given to parents.

Thirty-eight autistic children [14 girls; 10.94 yrs. (4.15)] and 34 typically developing peers [20 girls; 9.68 yrs. (2.75)] (all white European, middle socioeconomic status) met the inclusion criteria and agreed to participate in the study. Table 1 displays the clinical characteristics of autistic paticipants. Parents signed written informed consents. The objective and activities of the study were verbally and visually explained to the children and all participants gave their oral approval to participate. The study protocol was approved by the Ethics Committee on Research from the University of the Balearic Islands (ref. 127CER19). Patient associations for autism were involved in developing the research questions and appropiate measurements, the study implementation and dissemination of findings among families. Participants were not compensated for participation.

**Table 1:** Clinical characteristics of autistic participants (*n* = 38).

Autistic participants	Clinical variable (*n*, percentage)
Level of severity	
Level 1	21 (55.26%)
Level 2	17 (44.74%)
Chronic pain	
Yes	7 (21.05%)
No	31 (78.95%)
Verbal ability	
Fluent communicative speech	22 (57.90%)
Speech with communicative	
Sentences but frequent echolalia	5 (13.15%)
A few communicative sentences	5 (13.15%)
A few words	6 (15.80%)
WISC-V scores (mean, SD)	
Composite score	91.44 (12.16)
Verbal comprehension	87.75 (11.59)
Visual spatial	94.00 (20.33)
Fluid reasoning	99.14 (14.43)
Working memory	86.29 (17.38)
Processing speed	90.33 (19.23)

n = number of participants.

### 2.2. Measures

#### 2.2.1. Somatosensory thresholds

Participants were individually assessed by an experienced investigator (IR). The investigator was unblinded for the diagnosis of the participant. Tactile, warm and cold detection, and pressure pain thresholds were determined bilaterally on the thenar eminence of the hand palms, according to the quantitative sensory testing protocol of Backonja et al. (2013). For each somatosensory modality threshold, the average scores of right and left hands were used for further statistical analyses. To avoid anxiety, at the start of the experimental session children were familiarized with the assessment procedure by using different stimuli in body locations other than the hand palm (e. g. hand dorsum, arm). All children understood and correctly followed the procedure and none of the participants expressed distress during the assessment. To avoid any bias due to autistic children not reporting pain, the child’s mother or father observed the child during the procedure to report possible signs of distress and stop the assessment. The total duration of the assessment was twenty minutes.

*Tactile thresholds* were measured with Von Frey monofilaments (Keizer et al., 2008), with a diameter ranging from 0.14 to 1.01 mm and a pressure force ranging from 1.7 to 137.3 g/mm^2^, according to the method of limits (Backonja et al., 2013). The assessment was performed by touching the skin in a perpendicular way, pressing the monofilament slowly down till it buckled, holding it steady during 1.5 s, and removing it in the same way as it was applied. After several practice trials, children were asked to close their eyes and tell if they felt any touch sensation by saying “yes” or “no.” Null stimuli (no touch) were applied to check for false positive responses. Responses with more than 3 s. delay were considered as undetected. Body sides were stimulated in a pseudorandomized order. The procedure started with a thick filament and depending on the participant’s detection, subsequent monofilaments were applied with increasing or decreasing diameters. The tactile detection threshold of each body side was determined as the thinnest filament identified by the participant in three subsequent assessments. This procedure has previously been used to assess tactile thresholds in autistic children (Riquelme et al., 2016, 2018).

*Thermal detection thresholds* were measured on the thenar palms with a computer-controlled contact thermal stimulator (Cold/warm plate AHP-301CPV, Teca, Schubert, IL, United States). Before starting the measurement of thresholds, baseline skin temperature was established for each participant, by increasing or decreasing temperature from 32°C (resting temperature of the skin), as described in other studies of temperature perception in children with ASD (Williams et al., 2019). For measuring the *warm detection thresholds*, the individual baseline temperature was increased at a mean rate of 1°C/s up to a maximum temperature of 50°C. For the measurement of *cold detection thresholds*, the baseline temperature was decreased at the same rate until a minimum temperature of 0°C. Participants were instructed to keep the skin in contact with the thermal plate and to report the first perception of warm and cold, respectively. This procedure has been used previously to assess thermal thresholds in autistic children (Williams et al., 2019).

*Cold pain thresholds* were measured at the same body locations with the same thermal stimulator. Participants were instructed to keep the skin in contact with the thermal plate and to retract the hand at the first sensation of pain. Cold pain thresholds were assessed in two different ways: (1) temperature was decreased from the non-painful baseline temperature at a mean rate of 1°C/s to a minimum temperature of 0°C; the cold pain threshold was considered as the temperature at which the participant removed the hand; and (2) the thermal plate was set at a constant temperature of 0°C and participants were instructed to keep the thenar palm in contact with the thermal plate base and to retract the hand when the cold sensation first became painful (maximal testing time: 180 s). The first method yielded a variable in degrees (°C). The second method yielded a variable in time (s). This procedure has previously been used to assess thermal pain thresholds in autistic children (Williams et al., 2019).

*Pressure pain thresholds* (expressed in Newtons) were measured with a digital dynamometer using a flat rubber tip (surface of the tip: 1 cm^2^). Participants were asked to say “pain” or to raise a hand when the pressure became painful and this was considered as the pressure pain threshold. Pressure was released when the pain threshold or maximally exerted pressure of the dynamometer was reached. Three pressure stimuli were applied randomly on each thenar palm. The reliability of this procedure for assessing pressure pain sensitivity has been demonstrated in previous studies in autistic children (Riquelme et al., 2016, 2018).

#### 2.2.2. Emotion and behavior assessments

##### 2.2.2.1. Emotion matching task

This task measures the emotion knowledge of happiness, sadness, anger and fear/surprise in children through four different tasks. In task EMT1 (expression matching), the child is shown a photograph with an emotion expression and is asked to choose a face with a similar emotion expression among four options. In task EMT2 (emotion situation knowledge), a specific situation is described (for example, “a child who has just been pushed”) and the child is asked to select among four facial expressions, the one that better represents that situation. In task EMT3 (expressive emotion knowledge), a facial expression is presented and the child is asked to define the emotion with a word. In task EMT4 (receptive emotion knowledge), an emotion concept is presented (for example, “tell me who is happy”) and the child is asked to identify the facial expression that corresponds to this situation. Each task consists of 12 items. The score of each task is computed by adding up the number of correct hits. The original version of the EMT presents high reliability, with α coefficients for the total test of 0.81–0.88 and 0.65, 0.54, 0.76 and 0.80, for each of the 4 tasks, respectively (Morgan et al., 2010). The Spanish version of the scale, with α coefficients between 0.82 and 0.94, was used in this study (Alonso-Alberca et al., 2012).

##### 2.2.2.2. Emotion regulation checklist

This questionnaire consists of 24 items, answered by a Likert scale of 4 points (never/almost never/always/almost always). The questionnaire provides information on two subscales: emotional lability/negativity and emotional regulation. This tool has reported a high reliability (emotional lability α = 0.96, emotional regulation α = 0.83, Shields and Cicchetti, 1997).

##### 2.2.2.3. Child behavior checklist

This is a questionnaire aiming at assessing social competence and behavior problems in children from 6 to 18 years. The present study used the part assessing behavior problems. It is composed of 118 items organized into 8 subscales that measure different factors involved in the development of children at the cognitive, behavioral, emotional and psychosomatic levels (i.e., anxious–depressed, withdrawn–depressed, somatic complaints, social problems, thought problems, attention problems, rule-breaking behavior and aggressive behavior). Some of these subscales can be grouped in two new dimensions: Internalizing problems (i.e., anxious/depressed, withdrawn/depressed, somatic complaints) and Externalizing problems (i.e., rule-breaker behavior, aggressive behavior). A score higher than 60 in the total score indicates psychopathology. This test has shown good predictive power, as well as providing high sensitivity, reliability (α = 0.95), discriminant power and utility at the transcultural level (Achenbach and Edelbrock, 1983).

##### 2.2.2.4. Short sensory profile

The sSP is a 38-item caregiver questionnaire that assesses sensory processing dysfunction in children and adolescents (Dunn, 1999; Beaudry-Bellefeuille and Lane, 2015). The 38 items are divided in 7 domains of sensory processing function (taste, tactile, smell, movement, visual and auditory sensitivity, and under-responsiveness/sensation seeking). Each item can be rated on a Likert scale of 5 points ranging from 1 (always) to 5 (never). Lower scores express more sensory reactive behaviors. This tool has good psychometric properties (Intraclass correlation = 0.88, *r* = 0.87) (McIntosh et al., 1999).

### 2.3. Statistical analysis

Data were anonymized and codified when building the database, to ensure blinding of the statistical process. Statistical analyses were performed using the SPSS software. Descriptive statistics were used to characterize demographic and clinical variables. Since Kolgomorov-Smirnov normality tests showed a near normal distribution for all variables (all *p* > 0.055), chi-square and t-tests were used for comparing variables between groups (autistic children versus typical developing peers). Two-way ANOVA with the between-subject factors GROUP (autistic participants vs. TDP) and SEX (boys vs. girls) were further performed for exploring gender differences in all variables. Pearson correlations were performed between somatosensory thresholds and emotional and behavior measures. Significance level was set at *p* < 0.05.

## 3. Results

**Figure 1:**
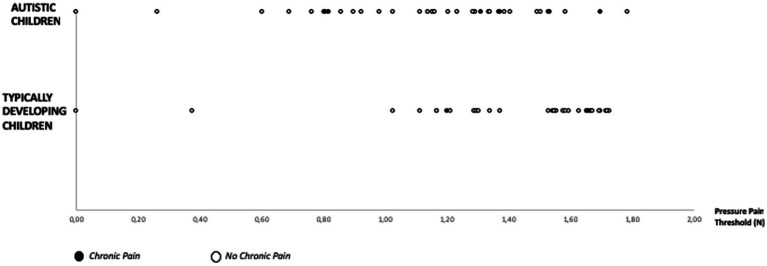
Scatterplot with descriptive data of chronic pain and pain pressure thresholds in autistic children and their typically developing peers. N = Newton.

Autistic children and adolescents were similar to the comparison group participants in age and sex. Autistic children and adolescents showed average scores (normative data) for all intellectual abilities, except for the Verbal Comprehension and Working Memory scales, where autistic children and adolescents showed lower scores than the normative data. Although chronic pain was slightly higher in autistic individuals (18.42%) than in their typically developing peers (TDP) (5.88%), there were no significant differences in prevalence rates between the groups (chi square (1,69) = 0.82, *p* = 0.151) nor between genders (chi square (1,69) = 0.30, *p* = 0.71). Figure 1 shows the individual pressure pain thresholds in each group of participants according to the presence of pain.

### 3.1. Somatosensory thresholds

Table 2 shows results for the somatosensory thresholds. Threshold values in TDP were consistent with those of prior research (Sampaio et al., 2019; Arnstad et al., 2020; Truffyn et al., 2021). Autistic children and adolescents had lower pressure pain thresholds than their TDP (*t* (1,69) = 2.51, *p* = 0.015). No significant differences between groups were found for tactile, warm detection, cold detection or cold pain thresholds. The relatioships between pressure pain thresholds and chronic pain for each group are displayed in Figure 1.

**Table 2:** Somatosensory thresholds in autistic children and their typically developing peers.

	Autistic children	Typically developing peers	
	Mean (SD)	Mean (SD)	Significance level
Tactile detection threshold (g/mm^2^)	0.4 (0.79)	1.1 (5.44)	0.415
Warm detection threshold (°C)	32.5 (5.02)	34.2 (5.54)	0.268
Cold detection threshold (°C)	26.2 (3.82)	28.0 (6.02)	0.205
Cold pain threshold (°C)	16.6 (23.62)	14.1 (6.00)	0.884
Cold pain threshold (in seconds at 0°C)	16.0 (35.71)	16.8 (7.77)	0.145
Pressure pain threshold (in Newton)	22.14 (20.07)	32.83 (14.89)	0.013

When analyzing differences between boys and girls, a significant interaction was found for cold pain thresholds (*F* (1,67) = 4.95, *p* = 0.030), indicating that autistic boys spent less time at 0°C (more pain sensitivity) than their TDP (*p* = 0.013). No significant differences were found between autistic girls and their TDP (*p* = 0.378). No other significant gender-related differences were found for somatosensory thresholds.

### 3.2. Emotion and behavior measures

Table 3 shows results of the emotion and behavior assessments. No significant differences between groups were found for the emotion knowledge task, or for the subscales of the Emotion Regulation Checklist (emotion regulation and emotion lability) (all *t* < 0.193, all *p* > 0.144). No significant gender-related differences were found for any of the emotion or behavior measurements.

**Table 3:** Emotion and behavior questionnaires in autistic children and their typically developing peers.

	Autistic children	Typically developing peers	
	Mean (SD)	Mean (SD)	Significance level
**Short sensory profile**			
Sensory reactivity	157.8 (27.918)	141.9 (28.16)	0.022
Tactile sensitivity	31.1 (4.37)	28.6 (5.57)	0.038
Taste/smell sensitivity	16.6 (5.12)	14.1 (6.00)	0.070
Movement sensitivity	13.3 (2.73)	11.5 (3.77)	0.026
Under-responsive	28.67 (6.22)	26.8 (6.48)	0.216
Auditory filtering	21.1 (6.95)	20.9 (5.71)	0.910
Low energy	26.5 (5.58)	22.5 (6.97)	0.011
Visual/auditory sensitivity	20.6 (4.84)	17.7 (4.83)	0.015
**Emotion Regulation Checklist**			
Emotion regulation	3.2 (0.65)	3.2 (0.75)	0.807
Emotion lability	1.7 (0.67)	1.8 (0.60)	0.673
**Child Behavior Checklist**			
Anxious–depressed	5.4 (3.87)	3.9 (4.23)	0.146
Withdrawn–depressed	4.8 (3.04)	1.9 (2.46)	0.001
Somatic complaints	1.7 (1.69)	0.8 (1.19)	0.026
Social problems	7.1 (4.47)	2.8 (2.76)	0.001
Thought problems	6.4 (4.56)	1.2 (1.65)	0.001
Attention problems	7.0 (3.31)	2.6 (2.47)	0.001
Rule-breaking behavior	4.5 (3.85)	2.1 (1.90)	0.003
Aggressive behavior	8.9 (6.30)	4.7 (5.71)	0.007
Externalizing problems	10.7 (7.49)	6.5 (6.68)	0.017
Internalizing problems	12.7 (9.05)	6.7 (7.04)	0.004

Autistic children and adolescents had higher scores than their TDP for the CBCL total score, internalising problems and externalising problems (all *t* > 2.45, all *p* < 0.017). Moreover, their scores were higher for all CBCL subscales (withdrawn–depressed, somatic complaints, social problems, thought problems, attention problems, rule-breaking behavior and aggressive behavior, all t > 2.25, all *p* < 0.028), except for the anxious-depressed subscale (*t* (1,62) = 1.47, *p* = 0.146). None of the results in autistic children were indicative for psychopathology.

Autistic children and adolescents had higher total scores for the Short Sensory Profile than their TDP (*t* (1,67) = 2.34, *p* = 0.022), indicating lower responsiveness to sensory stimuli. Autistic children and adolescents scored higher than their TDP for tactile sensitivity, movement sensitivity, low energy and visual–auditory sensitivity (all *t* > 2.11, all *p* < 0.038), whereas no significant differences between groups were found for taste–smell sensitivity, under-responsiveness and auditory filtering (all *t* < 1.24, all *p* > 0.070).

### 3.3. Correlations between somatosensory thresholds, emotion and behavior measures

**Figure 2:**
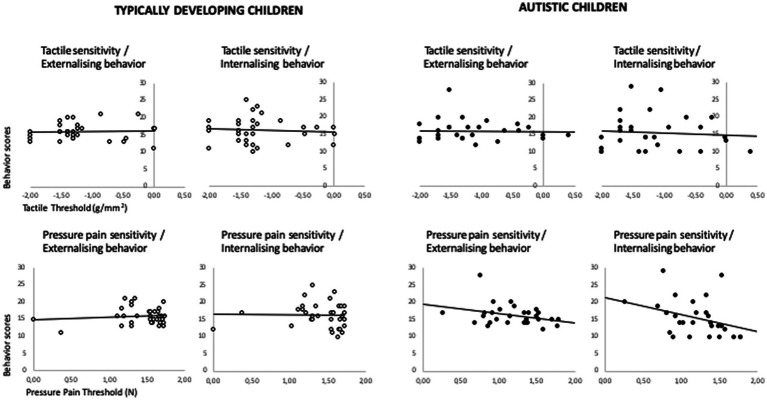
Scatterplots of correlations between tactile and pressure pain thresholds and internalising and externalising behavior in autistic children and typically developing peers. Sensitivity thresholds are displayed in log10 values; negative values showing scores lower than 1.

Correlations were performed separately for each group. In TDP, tactile thresholds were correlated with EMT1 (*r* = −0.538, *p* = 0.002), indicating that children with higher tactile sensitivity performed better when matching emotional facial expressions. In autistic children and adolescents several significant correlations were observed between somatosensory thresholds, emotion and behavior measures. Cold pain thresholds (measured in seconds) were correlated with emotion regulation (*r* = −0.532, all *p* < 0.002), suggesting lower emotion regulation in children with higher cold pain sensitivity. Tactile sensitivity and pressure pain thresholds were correlated with externalizing behavior problems (all *r* > −0.345, all *p* < 0.045), suggesting greater behavior problems in children with higher tactile and pressure pain sensitivity (Figure 2).

## 4. Discussion

The present study aimed at investigating the relationship between skin-mediated somatosensory signals and emotion/behavior difficulties in autistic children and adolescents, in comparison with typically developing peers (TDP). Results indicated that autistic children had higher pain sensitivity, less sensory reactive behaviors and more behavior problems than their TDP. In autistic children, skin-mediated hyper-sensitivity was correlated with emotion regulation and behavior problems. These findings confirm the importance of the relationship between abnormal skin-mediated somatosensation and emotion and/or behavior problems in autistic children.

The present results confirm the existence of abnormal sensation and behavior in autistic children, as reported in recent publications (Kirby et al., 2022; Samanta et al., 2022). The multidimensional evaluation in the present study revealed an interesting distinction between psychophysical thresholds (as assessed by quantitative sensory testing) and parental observations of sensitivity-related behaviors (as assessed by the short Sensory Profile). Through the sSF, parents reported hyposensitivity for many types of sensory stimuli (tactile, visual, low energy, movement) in their autistic children compared to a normal sensitivity in TPD. These results are in line with previously published data in autistic children and TDP (Dubourdieu and Guerendiain, 2022). In contrast, psychophysical thresholds indicated an increased sensitivity for pressure pain, and normal thresholds for temperature stimuli and non-painful tactile stimuli in autistic children. This discrepancy between heightened skin-mediated sensivitiy and diminished behavioral sensory sensitivity has been described previously in pain research in individuals with autism. These studies attributed the parent-reported behavioral hyposensitivity to a misled interpretation of poor pain expression or to poor pain responsivity due to social communication difficulties (Allely, 2013; Failla et al., 2020a). Furthermore, and in line with previous research (Williams et al., 2019; Fanghella et al., 2022), the findings of the present study highlight that affective situations of daily life (such as those assessed in questionnaires) and laboratory assessments (such as perceptive somatosensory thresholds) explore different functional aspects of brain networks that process sensory perception and interaction in autistic children. Further studies should investigate how factors such as emotion, attention and social communication could potentially modulate sensory perception in autism.

Quantitative sensory testing is a valuable tool to test the functional status of specific somatosensory channels by applying stimuli with predetermined physical characteristics. It is frequently used in pain research. There is a large interindividual variability of normative data, especially for pain thresholds (Rolke et al., 2006; Sobeeh et al., 2023). This compromises the interpretation of test results at a single time point for a given individual. However, quantitative sensory testing measurements are of value for group comparisons in research, such as used in the present study (Backonja et al., 2013). The results of this study revealed pain hypersensitivity in autistic children. Also, though statistically unsignificant, the number of children with chronic pain was higher in the group of autism than in TDP. Other studies have also reported aberrant pain processing (Riquelme et al., 2016, 2018; Gu et al., 2018) and frequent presence of chronic pain in autistic individuals (Bogdanova et al., 2022). Hypersensitivity has been related with sensitisation of the central nervous system, which would make autistic individuals especially prone to central sensitivity conditions, such as chronic pain (Grant et al., 2022). Though pain may be an important issue to address in autistic individuals, research in pain-relieving rehabilitation strategies is scant. Behavioral-based educational interventions have proven to be effective in increasing pain communication in autistic children with intellectual disability (Fitzpatrick et al., 2022). Somatosensory training of touch, proprioception, vibration and stereognosis has shown to reduce pain sensitivity in autistic children (Riquelme et al., 2018). Further research in this important topic is warranted.

Although autistic children and adolescents generally presented with somatosensory thresholds similar to those of the comparison group, these sensitivity measures were relevant, since they revealed significant relationships with emotional and behavioral aspects. Thus, our findings seem to indicate that the relationship between sensitivity to bodily signals and emotional/behavioral regulation is more relevant in autistic children than in typically developing children. For example, it has been suggested that somatosensory perception and higher order processing could be considered unique mechanisms that contribute to impaired emotional regulation in autistic people (Mazefsky et al., 2013; Bennett et al., 2017). In addition, it has been observed that in autistic children there is a close relationship between the abnormal perception of other bodily signals, such as interoception, and emotional and behavioral regulation problems, such as anxiety in social situations (Wood et al., 2022; Ben Hassen et al., 2023). The present concept of interoception also considers the skin as a sensory organ that can contribute to monitoring the physiological state of the body (Crucianelli and Ehrsson, 2023), and therefore skin-mediated signals such as touch, pain, and temperature could play an essential role in the construction of body consciousness (Failla et al., 2020b). It has been shown that the perception of bodily signals facilitates the use of emotional regulation strategies and the flexible selection of the appropriate one for a given situation in neurotypical individuals (Tan et al., 2023). Given that deficits in the modulation of emotional states have been related to internalizing and externalizing disruptive behaviors in healthy and psychopathological populations (Supplee et al., 2009; Cai et al., 2021; Njardvik et al., 2022), it seems to be plausible that deficits in sensory processing may be involved in the development of behavioral disorders in autistic individuals (Sung et al., 2022). Furthermore, alexithymia (the difficulty of describing and identifying one’s own emotions) seems to play a prominent role in the relationship between interoception and emotion regulation, and appears to be unrelated to co-occurring autism. Alexithymic traits are associated with a reduced use of interoceptive cues, reduced interoceptive accuracy and somatoform disorders both in autistic individuals and TDP (Murphy et al., 2018; Zdankiewicz-Scigala et al., 2021). The co-occurrence of autism and alexithymia seems to increase the interdependence of interoception and emotion regulation. For instance, a study in autistic adults revealed impaired interoception related to emotional clarity and alexithymia, while in TDP interoception only was associated with alexithymia (Bonete et al., 2023), an accurate assessment of alexithymia could lead to recognizing distinct groups of autistic children with distinct difficulties. For example, interoceptive awareness of emotions in facial expressions could be only impaired in autistic individuals with high levels of alexthymia (Butera et al., 2023). Further research should clarify the role of alexithymia in sensory and interoceptive processing, and its assessment should be incorporated in the rehabilitation process for characterizing autistic children and tailoring the intervention, in line with a person-based therapeutic approach.

When assessing the relationship between somatosensation and emotion/behavior regulation, the present data showed that the TPD group only exhibited a significant link between tactile sensitivity and emotional facial expression recognition. This was the only significant relationship that was evidenced. Thus, unlike autistic individuals, sensory processing did not appear to be an important factor affecting emotion regulation and behavior in typically developing children. Distinct factors may contribute with different weights to the development of emotion regulation and social behavior in autistic children compared to their TDP. Body awareness has been described as a product of perception, integration, feedforward and feedback processing of sensorimotor signals combined with prior body knowledge and previous experience (Holly, 2020; Grechuta et al., 2021). In this context, the connectivity of neural networks would make it possible to interpret the meaning of sensory stimuli as physiological changes originated within a specific global complex environment, which would imply the integration of both sensory and cognitive signals for a correct construction of the emotional response (Holly, 2020). Moreover, cognitive processes would allow integrating emotional responses into decision-making and behavior (Shah et al., 2016). For instance, decision-making under uncertainty is associated with body awareness and emotion regulation in the neurotypical population. This is less the case in autistic individuals (Shah et al., 2016). It could be hypothesized that the sensori-cognitive integration processes differ between autistic children and adolescents, and typically developing peers.

## 5. Limitations

This research protocol relied on a multidimensional assessment combining psychophysical testing, self-performed tasks and parent-based questionnaires. These assessments documented the sensory, emotional and behavorial profile of the individual without giving information on the underlying mechanism or anatomical site of dysfunctioning in the neuraxis. Functional neuroimaging could be of interest to provide complementary information on how neural networks process sensory integration and emotion regulation in autistic children versus TDP. Stroking touch was not explored and should be included in future studies for further investigation of the relationships between affective touch and social interaction. The emotion knowledge task used static pictures, which could have been poorly representative of dynamic interpersonal interactions in natural environments. The co-occurrence of alexithymia, co-existing diagnoses, medication or the type of chronic pain were not taken into account in the present study. These factors could influence interoception and should be included in future investigations. Children and adolescents with level 3 of autism were excluded from recruitment; the present findings may only apply to subpopulations with levels 1 and 2 of autism.

## 6. Conclusion

In conclusion, sensory dysfunction may affect the development of emotional processing and behavior in autistic children and adolescents. Understanding the mechanisms of sensory processing may provide important clues to understanding co-occurring alterations in emotion and behavior brain networks in autistic children. This knowledge can lay the foundation for the implementation of early interventions with the aim of promoting regulated and adjusted behaviors in autistic children and adolescents. Some rehabilitation interventions such the ‘Interoception curriculum’ at school (Mahler et al., 2022), sensory garments (Lawson et al., 2022) and somatosensory training that includes massage, joint compression, brushing, etc. (Bodison and Parham, 2018; Riquelme et al., 2018), have shown effectiveness in modifying interoception awareness and somatosensory thresholds, and have improved emotion regulation and participation. Promoting early interventions that include sensory rehabilitation therapy in autistic children, may lead to a more adequate development of skin-related signal processing and interoception and a reduction of abnormalities in emotion and behavior upon adult life.

## Data availability statement

The raw data supporting the conclusions of this article will be made available by the authors, without undue reservation.

## Ethics statement

The studies involving human participants were reviewed and approved by Ethics Committee on Research from the University of the Balearic Islands (ref. 127CER19). Written informed consent to participate in this study was provided by the participants’ legal guardian/next of kin.

## Author contributions

IR and PM contributed to conception and design of the study. IR organized the database, performed the statistical analysis and wrote the first draft of the manuscript. All authors contributed to the article and approved the submitted version.

## Funding

This research was funded by MCIN/AEI/10.13039/501100011033, Spain, grant number PID2020-114967GA-I00.

## Conflict of interest

The authors declare that the research was conducted in the absence of any commercial or financial relationships that could be construed as a potential conflict of interest.

## Publisher’s note

All claims expressed in this article are solely those of the authors and do not necessarily represent those of their affiliated organizations, or those of the publisher, the editors and the reviewers. Any product that may be evaluated in this article, or claim that may be made by its manufacturer, is not guaranteed or endorsed by the publisher.

## Footnotes

^1^ www.jotform.com
